# Real-world outcomes with rituximab vs. conventional therapy in pemphigus vulgaris: a single-center Romanian cohort

**DOI:** 10.3389/fmed.2025.1691897

**Published:** 2025-11-12

**Authors:** Daciana Elena Brănișteanu, Torello Lotti, Cristina Colac Boțoc, Antonia Elena Huțanu, Cătălina Anca Munteanu, Roxana Paraschiva Ciobanu, Daniel Constantin Brănișteanu, Alin Gabriel Colac, Cătălina Ioana Onu Brănișteanu, George Brănișteanu, Ștefan Vasile Toader, Mihaela Paula Toader

**Affiliations:** 1 Grigore T. Popa University of Medicine and Pharmacy, Iasi, Romania; 2 University G. Marconi of Rome-Dermatology and Venereology, Rome, Italy; 3 University Clinical Railways Hospital, Iasi, Romania; 4 Institute for Cardiovascular Diseases C.C. Iliescu, Bucharest, Romania; 5 Recovery Hospital, Orthopedy Clinic, Iasi, Romania

**Keywords:** pemphigus vulgaris, rituximab, real-world study, corticosteroids, immunosuppressants

## Abstract

**Background:**

Pemphigus vulgaris (PV) is a rare but potentially life-threatening autoimmune blistering disease. Rituximab has recently gained prominence as a first-line treatment for moderate-to-severe PV, yet real-world evidence from Eastern Europe remains limited. This study compares clinical outcomes of rituximab versus conventional corticosteroid-based therapy in a Romanian PV cohort.

**Methods:**

We conducted a retrospective, single-center observational study including 17 patients diagnosed with PV between January 2021 and July 2025 in Iași, Romania. All patients initially received systemic corticosteroids with azathioprine or mycophenolate mofetil when indicated. Rituximab became available through the national reimbursement program in June 2024 and was prescribed for refractory or moderate-to-severe disease. Clinical outcomes assessed included time to disease control, remission and relapse rates, cumulative corticosteroid exposure, and adverse events.

**Results:**

Eight patients received rituximab and nine received conventional therapy. Rituximab led to faster disease control, with 100% of patients achieving control within 12 months compared with 55.6% in the conventional group. Complete remission at last follow-up was higher in the rituximab group (75%) than in the conventional group (44.4%). No relapses occurred in rituximab-treated patients during follow-up, whereas all patients treated conventionally experienced relapses (34 episodes in total). The cumulative corticosteroid dose was higher in the rituximab group (median 19.5 g vs. 15.5 g) due to prior exposure before therapy switch. Adverse events differed by treatment: rituximab was associated primarily with early infusion-related reactions and infections, while conventional therapy caused steroid-related toxicities.

**Conclusion:**

In this real-world cohort, rituximab demonstrated superior disease control and relapse prevention compared with conventional therapy. Despite limited follow-up, findings support earlier use of rituximab in PV management to reduce relapse burden and long-term corticosteroid exposure. Broader access to rituximab and improved diagnostic resources could meaningfully improve outcomes in resource-limited settings.

## 1 Introduction

Pemphigus vulgaris (PV) is a rare, potentially life-threatening autoimmune blistering disorder and the most common form of autoimmune bullous dermatosis, characterized by mucocutaneous lesions due to IgG autoantibodies targeting desmoglein (Dsg) 3, often with concurrent Dsg1 reactivity (1). Autoantibodies against Dsg3 primarily affect mucosal epithelia, whereas combined Dsg1/Dsg3 reactivity produces both mucosal and cutaneous involvement, consistent with the desmoglein compensation theory (2). In addition to anti-Dsg1 and anti-Dsg4 antibodies, non-desmoglein (non-Dsg) autoantibodies targeting desmocollins, muscarinic and nicotinic acetylcoline receptors, plakins, and mitochondrial proteins have been implicated in PV pathogenesis. These antibodies contribute to keratinocyte detachment and acantholysis through complementary signaling mechanism, supporting a multifactorial autoimmune model rather than a purely a desmoglein-driven process (3).

The global incidence is approximately 3 cases per million person-years, with regional variation from 0.1 to 5 per 100,000 annually. Higher prevalence is reported in the Middle East, the Mediterranean basin, and among Ashkenazi Jewish populations. PV typically presents in middle adulthood (mean age 36–60 years) with a slight female predominance (~1.5:1). Strong genetic associations include HLA- DRB*104:02 and HLA-DQB1*03:02. Before the advent of effective immunosuppression, mortality reached 70–90%, but has declined to 5 − 15% in contemporary series (4, 5).

Clinically, PV most often begins on mucosal surfaces, particularly the oral cavity, with flaccid bullae that rupture to form painful erosions. These lesions impair oral intake, may lead to malnutrition, and can involve any mucosal site. Cutaneous lesions typically present as vesicles, erosions, and flaccid bullae on erythematous or apparently normal skin, healing with post-inflammatory hyperpigmentation but without scarring (6).

Diagnosis relies on the integration of clinical, histopathological, and immunological findings. Histology demonstrates suprabasal acantholysis with the “tombstone” appearance of basal cells (7). Direct immunofluorescence (DIF), the gold standard, reveals intercellular IgG and complement C3 deposits in a net-like pattern (8). Enzyme-linked immunosorbent assay (ELISA) detects circulating anti-Dsg1 and anti-Dsg3 antibodies with high sensitivity and specificity, while indirect immunofluorescence (IIF) provides additional confirmation (9, 10). Although PV is primarily a mucocutaneous disease, elevated inflammatory markers such as C-reactive protein (CRP), erythrocyte sedimentation rate (ESR), neutrophil-to-lymphocyte ratio (NLR), and pro-inflammatory cytokines have been reported, correlating with disease severity without consistent systemic organ involvement (11, 12). Disease severity is routinely assessed using validated scoring systems such as the Pemphigus Disease Area Index (PDAI) and the Autoimmune Bullous Skin Disorder Intensity Score (ABSIS), which standardize the evaluation of cutaneous and mucosal involvement, guide treatment decisions, and monitor response (13).

Management aims to achieve durable disease control while minimizing treatment-related morbidity. Systemic corticosteroids remain a cornerstone of therapy, often combined with immunosuppressants such as azathioprine or mycophenolate mofetil as steroid-sparing agents. The therapeutic landscape has evolved over the past decade, with several international guidelines now recommending rituximab, an anti-CD20 monoclonal antibody, as a first-line option for moderate-to- severe PV, given its ability to induce higher remission rates, reduce relapses, and lower cumulative corticosteroid exposure. Cyclophosphamide and other agents are generally reserved for refractory disease due to their toxicity profiles (14–16). However, despite promising clinical trial results, evidence on the comparative effectiveness and safety of rituximab versus conventional corticosteroid-based regimens remains limited in real-world practice, particularly regarding long-term follow-up.

This study aimed to compare clinical outcomes, time to disease control, remission duration, relapse rates, corticosteroid exposure, and adverse events in PV patients treated with rituximab versus conventional regimens in a real-world setting.

## 2 Materials and methods

We conducted a retrospective, single-center observational study at the Dermatology Clinic of the Clinical Railway Hospital, Iași, Romania. The study included consecutive patients diagnosed with PV between January 2021 and July 2025.

Inclusion criteria:

1. Clinical features: mucocutaneous flaccid bullae and erosions;
2. Histopathology: suprabasal acantholysis with “tombstone” basal cells;
3. Serology: positive anti-Dsg 1 and/or Dsg3 antibodies by ELISA.

Although DIF is considered the diagnostic gold standard, it was not available for all patients; in such cases, the diagnosis was established according to International Pemphigus Committee (IPC) consensus criteria based on concordant clinical, histopathological, and serological findings.

Exclusion criteria:

1. Absence of confirmatory histopathological findings;
2. Negative or unavailable ELISA for anti-Dsg1 and anti-Dsg3 antibodies;
3. Isolated mucosal or cutaneous lesions without compatible histology or serology;
4. Incomplete medical records preventing confirmation of the diagnosis by at least two of the three domains (clinical, histopathological, serological).

All patients initially received conventional therapy with systemic corticosteroids (prednisone-equivalent 0.7–1.0 mg/kg/day, tapered as tolerated) with or without azathioprine (50–100 mg/day) or mycophenolate mofetil (2 g/day). Rituximab became available for PV in Romania in June 2024 and was prescribed to patients with uncontrolled disease after ≥3 weeks of adequate conventional therapy. The regimen was 1 g intra-venously on day 1 and day 15, followed by maintenance every 6 months.

Patients controlled on conventional therapy alone formed the comparator group. Rituximab (generic name), administered either as MabThera® (F. Hoffmann-La Roche Ltd., Basel, Switzerland) or Rixathon® (Sandoz International GmbH, Holzkirchen, Germany), was available through the national healthcare program. In line with reimbursement policies of the Romanian National Health Insurance House, biosimilar rituximab (e.g., Rixathon®) was preferentially prescribed over the originator product (MabThera®).

Transition from conventional therapy to rituximab was performed in accordance with the Romanian National Health Insurance treatment protocol for pemphigus vulgaris, which specifies eligibility based on disease severity, treatment failure, or intolerance to standard systemic therapy.

For each patient, demographic data, disease duration, clinical subtype, comorbidities, baseline Pemphigus Disease Area Index (PDAI), Autoimmune Bullous Skin Disorder Intensity Score (ABSIS), Dermatology Life Quality Index (DLQI), treatment details, time to disease control, remission status and duration, relapse frequency, and adverse events were recorded. Outcomes followed IPC definitions; relapse was ≥3 new lesions in 1 month not healing within 1 week, or extension of existing lesions after disease control.

Adverse events were defined as new clinical events emerging after treatment initiation. Events already present at baseline (e.g., infected bullous erosions) were recorded separately and not considered treatment-emergent.

Clinical outcomes were defined according to IPC consensus criteria. Disease control was defined as the absence of new or established lesions that did not heal spontaneously within 1 week, with most existing lesions showing healing. Complete remission was defined as the absence of new or established lesions for at least 2 months while the patient was receiving minimal therapy (prednisone ≤10 mg/day or equivalent) or no therapy. Partial remission was defined as the presence of transient new lesions that healed within 1 week while the patient was receiving minimal therapy or no therapy. Duration of remission was calculated from the date of first documentation of remission until relapse or last follow-up. Time to outcome (disease control, partial remission, and complete remission) was measured from treatment initiation to the first occurrence of the respective state, regardless of subsequent relapse or disease progression. Relapse was defined as the appearance of three or more new lesions within 1 month that did not heal within 1 week, or the extension of established lesions after disease control.

Clinical data were extracted retrospectively from standardized electronic medical records using a predefined data collection sheet. Disease severity scores (PDAI, ABSIS, DLQI) and clinical outcomes were assessed according to IPC consensus definitions. Two investigators independently reviewed medical records to ensure data accuracy; discrepancies were resolved by consensus with a senior dermatologist. As this was a retrospective study, blinding was not applicable.

There were no missing data for primary or secondary clinical outcomes, and complete-case analysis was therefore performed.

## 3 Results

### 3.1 Patient demographic data

Baseline characteristics of all 17 patients are summarized in Table 1. The study included 17 patients, with a mean age at diagnosis predominantly between 50 and 59 years (*n* = 7/17, 41.2%), followed by 40–49 years (*n* = 4/17, 23.5%) and 60–69 years (*n* = 4/17, 23.5%). Only one patient (*n* = 1/17, 5.9%) was diagnosed at 30–39 years, and one (*n* = 1/17, 5.9%) was over 70 years at diagnosis. Regarding sex distribution, 10 patients (58.8%) were male and 7 (41.2%) were female.

**Table 1:** Demographic and clinical characteristics of the study cohort.

Characteristics	Subgroup	*n* = 17	%
Age at diagnosis	30–39	1	5.9
	40–49	4	23.5
	50–59	7	41.2
	60–69	4	23.5
	>70	1	5.9
Sex	Male	10	58.8
	Female	7	41.2
Disease duration at the time of treatment initiation	< 1 year	10	58.8
	>1 year	7	41.2

At the time of treatment initiation, 10 patients (58.8%) had a disease duration of less than 1 year, while 7 patients (41.2%) had been diagnosed for more than 1 year.

### 3.2 Clinical presentation, disease severity, and comorbidities

Table 2 summarizes the baseline clinical characteristics of the cohort. Among the 17 patients, 41.2% (*n* = 7/17) had mucosal PV, 5.9% (*n* = 1/17) had cutaneous PV, and 52.9% (*n* = 9/17) presented with the mucocutaneous type. At baseline, no patients had mild disease by PDAI, DLQI, or ABSIS. By PDAI, 52.9% (*n* = 9/17) had moderate disease severity, and 47.1% (*n* = 8/17) had severe disease. The DLQI score showed no patients with mild-to-moderate impact, while 47.1% (*n* = 8/17) had high impact and 52.9% (*n* = 9/17) had extremely high impact. According to the ABSIS, none were classified as mild, 52.9% (*n* = 9/17) were moderate, and 47.1% (*n* = 8/17) were severe. Regarding diagnostic delay, 47.1% (*n* = 8/17) were diagnosed within 6 months of symptom onset, 35.3% (*n* = 6/17) between 6 months and 1 year, and 17.6% (*n* = 3/17) after more than 1 year.

**Table 2:** Clinical characteristics, baseline severity, diagnostic delay, and comorbidities of the 17 patients.

Characteristic	Subgroup	Score interpretation (when applicable)	*n* = 17	%
Type of PV	Mucosal		7	41.2
	Cutaneous		1	5.9
	Mucocutaneous		9	52.9
Baseline severity	PDAI	Mild (0–15 points)	0	0
		Moderate (15–45 points)	9	52.9
		Severe (>45 points)	8	47.1
	DLQI	Mild–moderate (<10 points)	0	0
		High impact (11–20 points)	8	47.1
		Extremely high (>20 points)	9	52.9
	ABSIS	Mild (0–16 points)	0	0
		Moderate (17–53 points)	9	52.9
		Severe (>54 points)	8	47.1
Time from symptom onset to diagnosis	<6 months		8	47.1
	6 months - 1 year		6	35.3
	>1 year		3	17.6
Presence of comorbidities at the time of diagnosis	Diabetes		5	29.4
	Hypertension		10	58.8
	Metabolic		5	29.5
	Psychiatric		2	11.7
	Neurological		3	17.6
	Autoimmune diseases		2	11.7

Comorbidities were frequent: hypertension was present in 58.8% (*n* = 10/17), diabetes in 29.4% (*n* = 5/17), metabolic disorders in 29.5% (*n* = 5/17), neurological conditions in 17.6% (*n* = 3/17), psychiatric conditions in 11.7% (*n* = 2/17), and concomitant autoimmune diseases in 11.7% (*n* = 2/17).

### 3.3 Histopathological and immunological findings

Table 3 presents the histopathological and immunological findings of the cohort. Histopathological confirmation was obtained in all patients (100%, *n* = 17/17). DIF testing was performed in 47.1% (*n* = 8/17) of cases, while 52.9% (*n* = 9/17) were diagnosed without it. Regarding serological markers, elevated anti-Dsg1 titers were found in 58.2% (*n* = 10/17) of patients, while 41.2% (*n* = 7/17) had normal levels. Anti-Dsg 3 titers were elevated in 94.1% (*n* = 16/17) of patients, with only 5.9% (*n* = 1/17) showing normal values.

**Table 3:** Histopathological, immunological, and serological characteristics of the 17 patients.

Investigations	Result	*n* = 17	%
Histopathology confirmation	Yes	17	100%
	No	0	0
Direct immunofluorescence	Yes	8	47.1
	No	9	52.9
Anti-desmoglein 1 titer	Normal	7	41.2
	Elevated	10	58.2
Anti-desmoglein 3 titer	Normal	1	5.9
	Elevated	16	94.1

### 3.4 Treatment regimens

Table 4 summarizes the treatment regimens administered in the cohort. All patients in the conventional therapy group were treated with systemic corticosteroids at baseline. The initial dose ranged from 0.7 to 1.0 mg/kg/day (prednisone-equivalent), adjusted according to individual comorbidities, and was subsequently tapered as tolerated. The median duration of corticosteroid therapy was 12 months, with a range from 8 months to 5 years. Among conventional adjuvant therapies, azathioprine was prescribed to eight patients. Of these, six received 50 mg/day and two received 100 mg/day, with a median treatment duration of 1 year (range, 8 months to 2 years). Mycophenolate mofetil was used in a single patient at a daily dose of 2 g for a duration of 2 months.

**Table 4:** Treatment regimens and cumulative corticosteroid dose in the 17 patients.

Treatment		Data
Rituximab (*n* = 8)		–1 g day 1, 1 g day 15, 1 g every 6 months;
		–median 3 cycles (range 1–3);
		–*n* = 6/8 patients received 3 cycles (75%),
		–*n* = 1/8 received 2 cycles (12.5%)
		–*n* = 1/8 received 1 cycle (12.5%)
Corticosteroids (*n* = 9)		–Initial dose: 0.7–1 mg/kg/day (adapted to comorbidities);
		–Progressive tapering;
		–Median duration: 12 months (range 8 months–5 years)
Azathioprine (*n* = 8)		−50 mg/day: *n* = 6/8
		−100 mg/day: *n* = 2/8
		–median duration: 1 year (range 8 months−2 years)
Mycophenolate mofetil (*n* = 1)		–2 g/day;
		–duration: 2 months
Concomitant betamethasone injections (in Rituximab group)		−1 dose (*n* = 6/8, 75%)
		−2 doses (*n* = 2/8, 25%)
Cumulative dose of corticosteroids	Rituximab group (*n* = 8)	Median 19.5 g (8.2–38.6 g)
	Conventional therapy group (*n* = 9)	Median 15.5 g (2.1–28.5 g)

Eight patients received rituximab following the national rheumatoid arthritis-like protocol: 1 g on day 1, 1 g on day 15, and 1 g every 6 months thereafter. The median number of cycles was three (range 1–3), with 75% (*n* = 6/8) receiving 3 cycles, 12.5% (*n* = 1/8) receiving 2 cycles, and 12.5% (*n* = 1/8) receiving a single cycle. In addition, concomitant intramuscular betamethasone injections were administered to 75% (*n* = 6/8) as a single dose and to 25% (*n* = 2/8) as two doses.

The median cumulative corticosteroid dose was 19.5 g (range 8.2–38.6 g) in the rituximab group and 15.5 g (range 2.1–28.5 g) in the conventional therapy group.

### 3.5 Treatments

Treatment outcomes are summarized in Table 5, with rituximab-treated patients reaching disease control more rapidly than those receiving conventional therapy. In the rituximab group, 62.5% (*n* = 5/8) achieved disease control within 6 months and 37.5% (*n* = 3/8) within 6–12 months, with no patients requiring more than 12 months. In contrast, in the conventional therapy group, 22.2% (*n* = 2/9) achieved disease control within 6 months, 33.3% (*n* = 3/9) within 6–12 months, and 44.4% (*n* = 4/9) required more than 12 months. Complete remission was reached in 37.5% (*n* = 3/8) of rituximab-treated patients within 6 months and 37.5% (*n* = 3/8) within 6–12 months, while 25% (*n* = 2/8) did not achieve this outcome during follow-up. In the conventional therapy group, 22.2% (*n* = 2/9) achieved complete remission within 6 months, another 22.2% (*n* = 2/9) within 6–12 months, and 55.6% (*n* = 5/9) did not achieve complete remission.

**Table 5:** Treatment outcomes, remission rates, and relapses in the rituximab and conventional therapy groups.

Outcome	Rituximab		Conventional therapies	
	*n* = 8	%	*n* = 9	%
Time to disease control				
<6 months	5	62.5	2	22.2
6–12 months	3	37.5	3	33.4
>12 months	0	0	4	44.4
Time to complete remission				
< 6 months	3	37.5	2	22.2
6–12 months	3	37.5	2	22.2
>12 months	0	0	0	0
Not achieved	2	25	5	55.6
Time to partial remission				
< 6 months	3	37.5	2	22.2
6–12 months	5	62.5	2	22.2
>12 months	0	0	5	55.6
Duration of complete remission				
< 6 months	0	0	1	11.1
6–12 months	6	75	3	33.4
>12 months	0	0	0	0
Not achieved	2	25	5	55.6
Duration of partial remission				
< 6 months	3	37.5	1	11.1
6–12 months	5	62.5	5	55.6
>12 months	0	0	3	33.3
Relapse rate				
Patients with relapse	No relapses observed during available follow-up		9	
Number of relapses	N/A	N/A	34	
Median number of relapse/ patient (only in relapsers)	N/A	N/A	4 (range 1–7)	
Median time to relapse	N/A	N/A	6 months (2–12)	

“Time to remission” refers to the first time each patient reached the respective remission state during follow-up, regardless of later disease course (e.g., some patients who first achieved complete remission may have relapsed or transitioned to partial remission by the last follow-up). Percentages are calculated based on the total number of patients in each treatment group.

Partial remission in the rituximab group was achieved in 37.5% (*n* = 3/8) within 6 months and in 62.5% (*n* = 5/8) within 6–12 months, with none requiring more than 12 months. In the conventional therapy group, 22.2% (*n* = 2/9) achieved partial remission within 6 months, 22.2% (*n* = 2/9) within 6–12 months, and 55.6% (*n* = 5/9) after more than 12 months. Regarding the duration of complete remission, 75% (*n* = 6/8) of rituximab-treated patients maintained remission for 6–12 months, while 25% (*n* = 2/8) never achieved it. In the conventional therapy group, 11.1% (*n* = 1/9) maintained complete remission for less than 6 months, 33.3% (*n* = 3/9) for 6–12 months, and 55.6% (*n* = 5/9) did not achieve remission. The duration of partial remission in the rituximab group was less than 6 months in 37.5% (*n* = 3/8) and 6–12 months in 62.5% (*n* = 5/8). In the conventional therapy group, 11.1% (*n* = 1/9) maintained partial remission for less than 6 months, 55.6% (*n* = 5/9) for 6–12 months, and 33% (*n* = 3/9) for more than 12 months.

In this small cohort, rituximab-treated patients reached disease control within 12 months, while conventional therapy patients more often required longer. No relapses were documented in the rituximab group during available follow-up, whereas relapses were observed in all patients on conventional therapy. The median number of relapses among relapsing patients was 4 (range 1–7), and the median time to first relapse was 6 months (range 2–12 months).

### 3.6 Adverse events

Tables 6, 7 summarize the adverse events observed in the rituximab and conventional therapy groups, respectively, highlighting distinct safety profiles between the two treatment strategies.

**Table 6:** Rituximab-related adverse events.

Outcome	Total		Onset classification			
			< 3 months		>3 months	
	*n* = 8	%	*n* = 8	%	*n* = 8	%
Infectious complications						
Interstitial pneumonia	3	37.5	3	37.5	0	0
Tinea unguium	2	25	0	0	2	25
Tinea corporis	3	37.5	0	0	2	25
Labial herpes simplex	1	12.5	1	12.5	0	0
Cardiovascular complications						
Hypotension	3	37.5	3	37.5	0	0
Atrial fibrillation	2	25	2	25	0	0
Ventricular extrasystoles	2	25	2	25	0	0
Respiratory complications						
Dyspneea	3	37.5	3	37.5	0	0
Pleural effusion	2	25	2	25	0	0
Neurological complications						
Vertigo	2	25	1	11.2	1	11.2

**Table 7:** Infectious and steroid-related adverse events in the conventional therapy group.

Outcome	Total		Onset classification			
	*n* = 9	%	<3 months		>3 months	
			*n* = 9	%	*n* = 9	%
Infectious complications						
Tinea unguium	2	22.2	0	0	2	0
Tinea corporis	1	11.1	0	0	2	0
Oral candidiasis	7	77.7	6	66.6	7	0
Bullous erosion superinfection	9	100	9	100	9	0
Labial herpes simplex	2	22.2	0	0	2	0
UTI	4	44.4	2	22.2	3	0
Others						
Weight gain	7	77.7	2	22.2	5	
Hyperglycemia	6	66.6	6	66.6	0	0
Iatrogenic Cushing syndrome	2	22.2	0	0	2	22.2
Hyperlipidemia	6	66.6	0	0	6	66.6
Osteoporosis	3	33.3	0	0	3	33.3
Steroid induced myopathy	5	55.5	0	0	5	55.5
Posterior subcapsular cataract	1	11.1	0	0	1	11.1
Hypertension	8	88.8	3	33.3	5	55.5
Fluid retention	9	100	5	55.5	4	44.4
Insomnia	6	66.6	5	55.5	1	11.1
Mood distrubance	8	88.8	5	55.5	3	33.3

In the rituximab group, most adverse events occurred within the first 3 months of treatment. Infectious complications included interstitial pneumonia in 37.5% of patients (*n* = 3/8), all of which developed early, while fungal infections such as tinea unguium (25%, *n* = 2/8) and tinea corporis (37.5%, *n* = 3/8) appeared later during follow-up. Labial herpes simplex was documented in 12.5% (*n* = 1/8), with early onset. Cardiovascular complications were observed in a substantial proportion, including hypotension in 37.5% (*n* = 3/8), atrial fibrillation in 25% (*n* = 2/8), and ventricular extrasystoles in 25% (*n* = 2/8), all occurring during the early phase of therapy. Respiratory adverse events included dyspnea in 37.5% (*n* = 3/8) and pleural effusion in 25% (*n* = 2/8), both exclusively early-onset. Neurological manifestations were less frequent, with vertigo reported in 25% (*n* = 2/8), occurring both early (*n* = 1/8) and later (*n* = 1/8) during treatment.

In the conventional therapy group, infectious complications were frequent. Oral candidiasis was reported in 77.7% of patients (*n* = 7/9), with most cases occurring within the first 3 months, and bullous erosion superinfection was universal (100%, *n* = 9/9), all of which developed early. Tinea unguium (22.2%, *n* = 2/9), tinea corporis (11.1%, *n* = 1/9), labial herpes simplex (22.2%, *n* = 2/9), and urinary tract infections (44.4%, *n* = 4/9) were also observed. Steroid-related adverse events were common and diverse. Weight gain was present in 77.7% of patients (*n* = 7/9), with both early (22.2%, *n* = 2/9) and late (55.5%, *n* = 5/9) onset. Hyperglycemia was reported in 66.6% (*n* = 6/9), exclusively within the first 3 months, while hyperlipidemia occurred in 66.6% (*n* = 6/9), osteoporosis in 33.3% (*n* = 3/9), steroid-induced myopathy in 55.5% (*n* = 5), and posterior subcapsular cataract in 11.1% (*n* = 1/9), all manifesting later in the course of treatment. Hypertension was detected in 88.8% (*n* = 8/9), with 33.3% early (*n* = 3/9) and 55.5% late onset (*n* = 5/9), while fluid retention was observed in all patients, more commonly during the first 3 months. Neuropsychiatric complications were also frequent, including insomnia in 66.6% (*n* = 6/9) and mood disturbances in 88.8% (*n* = 8/9), with most cases arising early in treatment.

### 3.7 Outcomes

As shown in Table 8, follow-up duration differed between groups, with rituximab-treated patients having shorter observation times due to later therapy introduction. The duration of follow-up differed between groups. In the rituximab group, 37.5% (*n* = 3/8) were followed for less than 1 year and 62.5% (*n* = 5/8) for 1–2 years, with no patients exceeding 2 years of follow-up. In contrast, in the conventional therapy group 44.4% (*n* = 4/9) were followed for 1–2 years and 55.6% (*n* = 5/9) for more than 2 years. At the last visit, complete remission was documented in 75% (*n* = 6/8) of rituximab- treated patients and 44.4% (*n* = 4/9) of conventionally treated patients, while partial remission was observed in 25% (*n* = 2/8) and 55.6% (*n* = 5/9), respectively. None of the patients in either group had active disease at the last assessment, and no cases were lost to follow-up.

**Table 8:** Duration of follow-up and disease status at last visit in PV patients treated with rituximab vs. conventional therapy.

Follow-up	Subgroup	Rituximab group		Conventional therapy group	
		*n* = 8	%	*n* = 9	%
Duration of follow-up after treatment	<1 year	3	37.5	0	0
	1–2 years	5	62.5	4	44.5
	>2 years*	-	-	5	55.5
Current disease status at last visit	Complete remission	6	75	4	44.5
	Partial remission	2	25	5	55.5
	Active disease	0	0	0	0
	Lost to follow-up	0	0	0	0

*No rituximab-treated patients had follow-up beyond 14 months after treatment initiation due to its late introduction in June 2024. **This table represents the patients’ status at the end of the observation period, which may differ from the remission timing data in Table 5 due to relapses or progression between remission categories.

## 4 Discussion

This retrospective observational study aimed to compare the clinical outcomes, remission profiles, relapse rates, corticosteroid exposure, and adverse events in patients with PV treated with rituximab versus conventional corticosteroid-based therapies in a real-world clinical setting.

Accurate diagnosis of PV relies on the integration of clinical, histopathological, and immunologic assessments. While DIF is considered the gold standard, its availability is often restricted to specialized centers due to the need for fresh perilesional tissue, specialized processing, and strict handling protocols. Prior systemic corticosteroid use can also suppress immune deposition, leading to false-negative DIF results. In our cohort, some patients were diagnosed without DIF confirmation, relying instead on characteristic clinical features, histopathologic suprabasal acantholysis, and positive anti-desmoglein ELISA serology (17). Although not all patients had access to DIF testing, the diagnosis was robustly established in every case through the integration of clinical features, histology, and serology where available. This ensured diagnostic accuracy even in the absence of uniform immunopathologic confirmation. Compounding these challenges, serological testing is often not reimbursed by the Romanian public healthcare system and must be self-funded, limiting routine use.

These findings highlight the need for pragmatic, resource-adapted diagnostic strategies in middle-income healthcare systems where immunopathology services and affordable serologic testing are not universally available.

A major challenge in PV management is diagnostic delay, particularly in mucosal-dominant cases (18). Oral erosions are frequently misdiagnosed as aphthous ulcers, erosive lichen planus, or infectious mucositis, delaying referral and timely immuno-pathological confirmation (19, 20). In our study, nearly half of patients presented with isolated mucosal disease, and over half experienced delays exceeding 6 months from symptom onset to diagnosis. This postponement of targeted immunosuppressive therapy allowed disease progression and extension to the skin, with nearly half of patients showing severe disease at baseline. All patients in our cohort had moderate-to-severe disease activity at presentation, minimizing the likelihood that outcome differences between groups were attributable to imbalances in disease severity. These findings align with prior studies reporting that diagnostic delay contributes to increased disease severity, higher cortico-steroid requirements, and risk of adverse events (21, 22). Early recognition of mucosal PV is therefore critical to improving outcomes and minimizing long-term treatment burden.

Management complexity further increases with patient age and comorbidities. In our cohort, the majority of patients were over 50 years old, and more than half had at least one major comorbidity, most commonly hypertension and diabetes. These conditions limited the use of high-dose systemic corticosteroids, requiring individualized dosing and close monitoring to balance disease control with the risk of metabolic, cardiovascular, and skeletal complications. Treatment heterogeneity within the rituximab group, including variation in the number of cycles received and the concomitant use of intramuscular betamethasone in some patients, may have influenced individual outcomes. However, this reflects real-world practice and the individualized adjustments often required in patients with multiple comorbidities. Similar findings have been reported in the literature, underscoring the need for multidisciplinary management in elderly PV patients, involving dermatologists, internists, endocrinologists, and cardiologists to anticipate and mitigate treatment-related harm (23, 24).

The high prevalence of comorbidities in our cohort, particularly hypertension and diabetes, may have influenced both treatment tolerance and safety outcomes. These conditions not only complicated corticosteroids administration but also introduced potential confounding factors in treatment response. Reporting comorbidity profiles is therefore essential to contextualize therapy outcomes in real-world practice, especially in elderly populations with multiple chronic conditions.

In our limited cohort, rituximab-treated patients appeared to achieve disease control more quickly, with earlier complete or partial remission, and tended to maintain remission longer compared with those receiving conventional therapies. Within the available follow-up, no relapses were documented among rituximab-treated patients, whereas relapses were common in the conventional therapy group, with a median of four episodes per patient. These observations should be interpreted cautiously given the small sample size and differences in follow-up duration between groups. However, cumulative corticosteroid exposure was paradoxically higher in the rituximab group, reflecting the fact that rituximab was only introduced in Romania in mid-2024 and could be prescribed exclusively after failure of adequate conventional therapy, in accordance with national protocol criteria. As such, patients eligible for rituximab had already accumulated significant corticosteroid doses before transitioning to biologic therapy. Follow-up duration was longer in the conventional therapy group, as rituximab only became an approved treatment option for PV in Romania in mid-2024. While this difference in observation time may partly explain the higher number of relapses captured in the conventional group, the absence of relapses in rituximab-treated patients remains clinically meaningful within the available follow-up. These observations are consistent with randomized trials and observational studies showing that rituximab reduces relapse rates, and achieves higher rates of durable remission (25, 26).

Our findings resonate with recent work by Scarpone et al., who reported that PV patients often require multiple therapy changes (median four per patient) to achieve disease control, reflecting the challenge of finding an effective, tolerable regimen (27). In contrast, rituximab-treated patients in both our study and prior reports needed fewer subsequent therapy modifications, highlighting the central role of B-cell depletion in PV pathogenesis. As expected, most mucosal-dominant patients in our cohort 327 had elevated anti-Dsg3 titers, while anti-Dsg 1 was more frequently associated with mucocutaneous involvement (28). Although our cohort size precluded formal statistical testing, these patterns are consistent with established pathogenic models. While anti-desmoglein antibody titers are valuable markers of disease activity, prior studies suggest they do not reliably predict treatment response or the need for therapy changes, reinforcing that serologic data should complement, not replace, clinical evaluation (29).

Beyond clinical effectiveness, the cost-effectiveness of rituximab has gained increasing attention. Cai et al. demonstrated that despite its higher upfront cost, rituximab was approximately 20% more cost-effective than conventional therapy regimens due to fewer hospitalizations, fewer relapses, and lower expenditures for managing corticosteroid and immunosuppressant-related complications (30). Our real-world observations align with these findings. Although formal healthcare resource use was not quantified, rituximab appeared to reduce the need for repeated treatment adjustments and hospital-based interventions, which may translate into lower long-term healthcare burden. In Romania, where healthcare budgets are limited, and where the trust of patients in the public medical system is often fragile, the ability of rituximab to reduce long-term treatment burden and improve outcomes carries added importance. These results suggest that evaluating therapeutic strategies should consider not only drug acquisition costs but also broader economic and health system impacts.

Complete responders in our cohort were typically patients with shorter disease duration, fewer or no comorbidities, excellent therapeutic compliance, and the ability to tolerate adequate immunosuppressant doses. In contrast, delayed diagnosis, misclassification of mucosal lesions, and the accumulation of comorbidities contributed to longer times to remission and greater cumulative corticosteroid exposure. Moreover, long-term corticosteroid use carries substantial risks, including metabolic derangements, bone loss, cardiovascular events, and infections, making cumulative dose monitoring essential. Adverse events in the rituximab group were largely early-onset, dominated by infusion-related cardiovascular and respiratory complications, while fungal infections emerged later during follow-up. In contrast, patients on conventional corticosteroid-based regimens experienced frequent infectious complications early in the course of therapy, followed by the cumulative development of metabolic, musculoskeletal, and ocular toxicities consistent with long-term steroid exposure. This temporal distinction highlights the different safety profiles of the two treatment strategies. It is important to interpret the adverse event data with caution. Because the number of patients in each group was small, percentages may exaggerate the apparent frequency of individual complications (e.g., interstitial pneumonia in three patients represented 37.5% of the rituximab group). Moreover, some events, such as superinfection of bullous erosions, are common complications of active PV itself and may not reflect treatment toxicity. For this reason, our tables present raw numbers alongside percentages, but quantitative comparisons between groups should be considered descriptive only.

In Romania, rituximab was only recently introduced as a therapeutic option for PV and can currently be prescribed only after national protocol approval, limiting routine access and contributing to the relatively limited local experience. Furthermore, DIF testing is restricted to a few specialized centers and is not routinely integrated into daily diagnostic workflows, while serologic testing often remains inaccessible to patients due to out-of-pocket costs. Despite these constraints, our findings align with international evidence and guideline recommendations positioning rituximab as a first-line therapy for moderate-to-severe PV. However, real-world implementation in middle-income settings such as Romania remains limited by access to immunopathology, high upfront treatment costs, and restrictive national approval protocols. To our knowledge, this is one of the first Romanian real-world cohorts evaluating rituximab in PV, demonstrating favorable clinical outcomes despite these healthcare system constraints. These pragmatic barriers highlight the urgent need for healthcare policy efforts to improve access to diagnostic and therapeutic resources, expand immunopathology services, streamline rituximab approval processes, and consider public reimbursement of serologic testing.

While our study focused primarily on clinical outcomes, it is important to note that faster remission and reduced relapse rates translate into meaningful improvements in patient quality of life. Mucosal lesions, nutritional compromise, pain, and visible skin erosions have profound effects on daily functioning, social interactions, and mental health. Incorporating patient-reported outcomes into future research will be essential to fully capture the holistic benefits of effective, steroid-sparing regimens and to inform patient-centered care models.

Although rituximab represents a higher upfront investment, it holds promise for reducing hospital admissions, preventing corticosteroid-induced complications, and lowering cumulative drug expenditures associated with repeated relapses. Evaluating the pharmacoeconomic impact of rituximab within Romanian healthcare settings is an important avenue for future research and could help inform policy decisions on funding and access. Early integration of rituximab into the treatment for PV may accelerate disease control, reduce cumulative corticoid exposure, and improve quality of life more rapidly by shortening the duration of active erosions, pain and functional impairment (25, 31). Compared to conventional immunosuppressants, rituximab also reduces treatment burden for both patients and healthcare services. Its intermittent infusion schedule requires fewer hospital visits and less frequent laboratory monitoring than azathioprine or mycophenolate mofetil, which often demand ongoing safety surveillance and dose adjustments. This simplified therapeutic course may improve adherence, reduce outpatient workload, and optimize long-term resource allocation in dermatology practice, particularly in resource-constrained healthcare systems.

To our knowledge, this is the first study reporting the clinical use of rituximab in pemphigus vulgaris by Romanian dermatologists. This novelty highlights the growing adoption of biologic therapies in Eastern Europe and provides locally relevant data that complement international experience.

The rapid and durable efficacy of rituximab demonstrated in randomized trials and real-world cohorts has already led major societies such as the European Academy of Dermatology and Venereology (EADV) and the British Association of Dermatologists to recommend rituximab as first-line therapy for moderate-to-severe pemphigus (15). As additional real-world evidence continues to confirm faster disease control and lower relapse rates compared with conventional immunosuppression, future guideline updates are likely to emphasize earlier initiation of rituximab, not only as rescue therapy but as part of standard care pathways.

Importantly, the integration of rituximab into everyday practice must be adapted to healthcare resource settings. In high-resource centers, rituximab may be incorporated through early induction protocols supported by therapeutic drug monitoring. However, in resource-limited settings, including parts of Eastern Europe, Latin America, and Asia, a tiered treatment algorithm may be more feasible- prioritizing rituximab for patients with severe disease, frequent relapses, or corticosteroid toxicity, while simultaneously expanding access to diagnostic tools such as DIF and anti-desmoglein ELISA (25, 26). National health systems could support this transition with simplified approval pathways, regional infusion programs, and bundled reimbursement policies, ensuring equitable access. Such context-sensitive implementation strategies would align clinical practice with international recommendations while maintaining economic feasibility.

This study has several limitations. The retrospective design, small sample size, and single- center setting limit the generalizability of our findings. The heterogeneity of follow-up durations and the absence of standardized outcome assessments may also have introduced variability. Additionally, some patients were diagnosed without DIF due to technical and logistic constraints, which, although reflective of real-world practice, may be considered a diagnostic limitation. Because rituximab became available only in mid-2024, patients had accumulated substantial corticosteroid exposure before switching. In addition, follow-up was shorter in the rituximab group, which may bias relapse detection. These factors further limit direct comparability between groups. Despite these challenges, this study provides valuable real-world insights into PV management and offers one of the earliest Romanian data sets on rituximab use. Future prospective, multicenter studies with larger cohorts are needed to validate these findings, assess long-term outcomes, refine patient selection criteria for rituximab, and quantify its pharmacoeconomic impact. Incorporating patient-reported outcomes and quality-of-life measures will be essential to capture the full impact of therapeutic strategies and guide personalized management approaches for PV.

## Data Availability

The original contributions presented in the study are included in the article/supplementary material, further inquiries can be directed to the corresponding author.

## References

[1] Balighi K Ashtar Nakhaei N Daneshpazhooh M Aryanian Z Aslani S Balighi S . Pemphigus patients with initial negative levels of anti-desmoglein: a subtype with different profile? Dermatol Ther. (2022) 35:e15299. doi: 10.1111/dth.15299, PMID: 34981632

[2] Sielski L Baker J DePasquale MC Attwood K Seiffert-Sinha K Sinha AA. Desmoglein compensation hypothesis fidelity assessment in pemphigus. Front Immunol. (2022) 13:969278. doi: 10.3389/fimmu.2022.969278, PMID: 36211362 PMC9537551

[3] Shrivastava P Mariam S Abid L Buch SA Ahmad SA Mansoori S . Rituximab in childhood and juvenile pemphigus vulgaris: a systematic review. Cureus. (2024) 16:e58288. doi: 10.7759/cureus.58288, PMID: 38752055 PMC11094568

[4] Rosi-Schumacher M Baker J Waris J Seiffert-Sinha K Sinha AA. Worldwide epidemiologic factors in pemphigus vulgaris and bullous pemphigoid. Front Immunol. (2023) 14:1159351. doi: 10.3389/fimmu.2023.1159351, PMID: 37180132 PMC10166872

[5] Liuqi Zhao Yan Chen Mingyue Wang. The global incidence rate of pemphigus vulgaris: a systematic review and meta-analysis. Dermatology. (2023) 239:514. doi: 10.1159/000530121 36944327

[6] Costan VV Popa C Hâncu MF Porumb-Andrese E Toader MP. Comprehensive review on the pathophysiology, clinical variants and management of pemphigus (review). Exp Ther Med. (2021) 22:1335. doi: 10.3892/etm.2021.10770, PMID: 34630689 PMC8495539

[7] Manocha A Tirumalae R. Histopathology of pemphigus vulgaris revisited. Am J Dermatopathol. (2021) 43:429–37. doi: 10.1097/DAD.0000000000001838, PMID: 33208597

[8] Di Lernia V Casanova DM Goldust M Ricci C. Pemphigus vulgaris and bullous pemphigoid: update on diagnosis and treatment. Dermatol Pract Concept. (2020) 10:e2020050. doi: 10.5826/dpc.1003a50, PMID: 32642305 PMC7319750

[9] Saleh AM El-Samanoudy SI Rashed LA Saleh MA. Evaluation of the pathogenicity of anti- desmoglein antibodies of pemphigus vulgaris patients using human organ culture assay. Arch Dermatol Res. (2020) 312:289–94. doi: 10.1007/s00403-019-01988-9, PMID: 31587106

[10] Van de Gaer O de Haes P Bossuyt X. Detection of circulating anti-skin antibodies by indirect immunofluorescence and by ELISA: a comparative systematic review and meta-analysis. Clin Chem Lab Med. (2020) 58:1623–33. doi: 10.1515/cclm-2019-1031, PMID: 32335537

[11] Mortazavi H Saeidi V Balighi K Esmaili N Teimourpour A Daneshpazhooh M . Serologic biomarkers in pemphigus monitoring: C-reactive protein, macrophage migration inhibitory factor, and prolactin levels versus autoantibody assays. Iran J Allergy Asthma Immunol. (2023) 22:312–8. doi: 10.18502/ijaai.v22i3.13059, PMID: 37524667

[12] Aiholli S Adya KA Inamadar AC. Role of hematological indices as predictors of systemic inflammation in dermatology. Indian Dermatol Online J. (2023) 15:188–95. doi: 10.4103/idoj.idoj_189_23, PMID: 38550825 PMC10969267

[13] Mohebi F Tavakolpour S Teimourpour A. Estimated cut-off values for pemphigus severity classification according to pemphigus disease area index (PDAI), autoimmune bullous skin disorder intensity score (ABSIS), and anti-desmoglein 1 autoantibodies. BMC Dermatol. (2020) 20:13. doi: 10.1186/s12895-020-00105-y, PMID: 33129291 PMC7603731

[14] Zhao W Wang J Zhu H Pan M. Comparison of guidelines for Management of Pemphigus: a review of systemic corticosteroids, rituximab, and other immunosuppressive therapies. Clin Rev Allergy Immunol. (2021) 61:351–62. doi: 10.1007/s12016-021-08882-1, PMID: 34350539

[15] Joly P Horvath B Patsatsi Α Uzun S Bech R Beissert S . Updated S2K guidelines on the management of pemphigus vulgaris and foliaceus initiated by the european academy of dermatology and venereology (EADV). J Eur Acad Dermatol Venereol. (2020) 34:1900–13. doi: 10.1111/jdv.16752, PMID: 32830877

[16] Vafaeian A Mahmoudi H Daneshpazhooh M. What is novel in the clinical management of pemphigus vulgaris? Expert Rev Clin Pharmacol. (2024) 17:489–503. doi: 10.1080/17512433.2024.2350943, PMID: 38712540

[17] Petit M Walet-Balieu ML Schapman D Golinski ML Burel C Barray M . Longitudinal pathogenic properties and N-glycosylation profile of antibodies from patients with pemphigus after corticosteroid treatment. Biomedicine. (2021) 9:1411. doi: 10.3390/biomedicines9101411, PMID: 34680528 PMC8533488

[18] Pranadwista ZF Rahayuningtyas ED Sufiawati I. Addressing challenges in diagnosis, differential diagnosis, and treatment of pemphigus: a case series. Diagnostics (Basel). (2023) 13:3633. doi: 10.3390/diagnostics13243633, PMID: 38132217 PMC10742496

[19] Thayalan D Krishnan R Annasamy R Nataraj P Indumathi N. Oral pemphigus vulgaris with one year follow-up and complete remission. Oral Oncol Rep. (2024) 10:100305. doi: 10.1016/j.oor.2024.100305

[20] Al-Harbawee A Kassam K Patel AN Cottom H Cheng L. Oral pemphigus vulgaris: Dentists take-home message. Clin Case Reports. (2021) 9:e04494. doi: 10.1002/ccr3.4494, PMID: 34267920 PMC8271215

[21] Lamichhane R Chaudhary S. Delayed diagnosis of pemphigus vulgaris initially presenting as an Oral ulcer: a case report. JNMA J Nepal Med Assoc. (2022) 60:641–3. doi: 10.31729/jnma.7594, PMID: 36705190 PMC9297360

[22] Chen AW Liu L Yang H Luo XY Xiang J. Clinical features, diagnosis and treatment of different types of paediatric pemphigus. Acta Derm Venereol. (2025) 105:adv42776. doi: 10.2340/actadv.v105.42776, PMID: 40059466 PMC11926551

[23] Tarakji B. Pemphigus vulgaris in old patient. Case Rep Dent. (2021) 2021:1–3. doi: 10.1155/2021/3946161, PMID: 34476107 PMC8408008

[24] Quintarelli L Coi A Maglie R Corrà A Mariotti EB Aimo C . Clinical patterns, survival, comorbidities, and treatment regimens in 149 patients with pemphigus in Tuscany (Italy): a 12-year hospital-based study. Front Immunol. (2022) 13:895490. doi: 10.3389/fimmu.2022.895490, PMID: 35880183 PMC9307892

[25] Hébert V Hamwi S Tancrède-Bohin E Quéreux G Pham-Ledard A Caux F . MALIBUL group optimizing pemphigus management with rituximab and short-term relapse predictors. JAMA Dermatol. (2025) 161:399–405. doi: 10.1001/jamadermatol.2024.6130, PMID: 39908046 PMC11800125

[26] Kridin K Ahmed AR. The evolving role of rituximab in the treatment of pemphigus vulgaris: a comprehensive state-of-the-art review. Expert Opin Biol Ther. (2021) 21:443–54. doi: 10.1080/14712598.2021.1874915, PMID: 33455475

[27] Scarpone R Francuzik W Worm M Heine G. Therapy changes during pemphigus management: a retrospective analysis. Front Med (Lausanne). (2020) 7:581820. doi: 10.3389/fmed.2020.581820, PMID: 33330538 PMC7732666

[28] Ishii K Yoshida K Stanley JR Yamagami J Amagai M Ishiko A. Pemphigus vulgaris and Foliaceus IgG autoantibodies directly block Heterophilic Transinteraction between Desmoglein and Desmocollin. J Invest Dermatol. (2020) 140:1919–1926.e7. doi: 10.1016/j.jid.2020.02.010, PMID: 32142800

[29] Genovese G Maronese CA Casazza G Corti L Venegoni L Muratori S . Clinical and serological predictors of relapse in pemphigus: a study of 143 patients. Clin Exp Dermatol. (2022) 47:98–106. doi: 10.1111/ced.14854, PMID: 34288016 PMC9290045

[30] Cai Y Liu Y Deng S Luo Y Deng L. Efficacy of rituximab in the treatment of pemphigus vulgaris: a meta-analysis. Altern Ther Health Med. (2024) 30:136–41. 38401108

[31] Joly P Maho-Vaillant M Prost-Squarcioni C Hebert V Houivet E Calbo S . First-line rituximab combined with short-term prednisone versus prednisone alone for the treatment of pemphigus (Ritux 3): a prospective, multicentre, parallel-group, open-label randomised trial. Lancet. (2017) 389:2031–40. doi: 10.1016/S0140-6736(17)30070-3, PMID: 28342637

